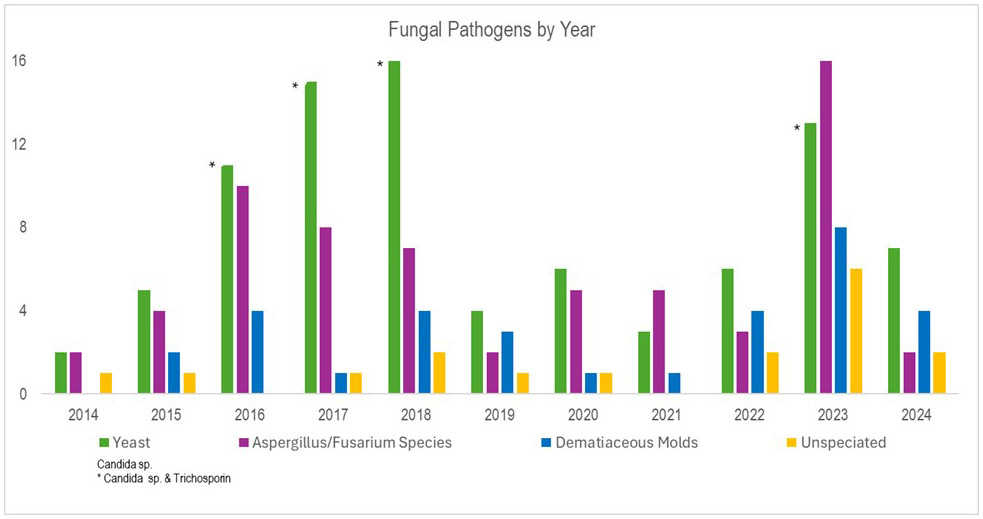# 866 A Decade of Fungal Infections from a Regional Burn Center

**DOI:** 10.1093/jbcr/iraf019.397

**Published:** 2025-04-01

**Authors:** Janie Faris, Courtney Rawitscher, Kandace Snodgrass, Rebecca Coffey, Samuel Mandell

**Affiliations:** Parkland Health Burn Center; University of Texas Southwestern Medical School; Parkland Health Burn Center; Parkland Health Burn Center; Parkland Health Burn Center

## Abstract

**Introduction:**

The incidence of fungal infections in burn patients is influenced by burn severity and fungal species. Rates of invasive fungal infections have been reported as high as 31% with mortality approaching 40%. Recently, the number of atypical fungal infections reported in the literature has been rising. This review describes the variation of fungal infections and outcomes of burn patients from one burn center over 10 years.

**Methods:**

Retrospective chart review of all patients admitted to a regional burn center from 2014-2024 who were treated for fungal infection. Patient characteristics, laboratory and microbiology culture data, medication administration records, and assessment of risk factors were evaluated. Outcomes were length of stay, fungal clearance, adverse events, and hospital mortality. Data analysis consisted of descriptive statistics (frequencies, median (IQR)).

**Results:**

94 patients were treated with systemic antifungals. 72% were male, 48% white, with a median age of 43. Thermal burns accounted for 80% of the injuries. Thirty percent had an inhalation injury, the median (IQR) TBSA was 47(40), ABSI score of 10(4.5), and rBAUX 94 (34.5). Known risk factors identified in this population included diabetes, chronic kidney disease, renal replacement therapy/dialysis, and previous antibiotic exposure. Candida species accounted for 38% of the total isolates, followed by Aspergillus/fusarium species 31%, Mucor/Rhizopus 7%, dematiaceous mold 9%, Trichosporin species 6% and unidentified species at 9%. Complications included: 30 VTE, 6 mycobacterial infections, 35 patients underwent amputations, and 3 patients experienced fungal brain abscesses. Voriconazole followed by liposomal amphotericin B were the most prescribed agents. Hospital and ICU LOS was 62(71) and 46(60) days, and duration of mechanical ventilation was 30(55) days. The hospital mortality rate was 44%.

**Conclusions:**

Most of the isolates were pathogens known to be difficult to treat or inherently resistant to multiple antifungals. The frequency of aspergillus species, fusarium and dematiaceous mold cultures vary over the study period but seem to have peaks that suggest intermittent outbreaks.

**Applicability of Research to Practice:**

Limited new antifungals and rapid diagnostic identification of fungal isolates is an area for future studies.

**Funding for the Study:**

N/A